# Prognostic significance of the modified Glasgow Prognostic Score in NSCLC patients undergoing immune checkpoint inhibitor therapy: a meta-analysis

**DOI:** 10.3389/fonc.2024.1449853

**Published:** 2024-10-11

**Authors:** Jiaxuan Wu, Haoyu Wang, Ruiyuan Yang, Dan Liu, Weimin Li

**Affiliations:** ^1^ Department of Pulmonary and Critical Care Medicine, West China Hospital, Sichuan University, Chengdu, Sichuan, China; ^2^ Institute of Respiratory Health, Frontiers Science Center for Disease-related Molecular Network, West China Hospital, Sichuan University, Chengdu, Sichuan, China; ^3^ The Research Units of West China, Chinese Academy of Medical Sciences, West China Hospital, Chengdu, Sichuan, China; ^4^ Institute of Respiratory Health and Multimorbidity, West China Hospital, Sichuan University, Chengdu, Sichuan, China; ^5^ Precision Medicine Center, Precision Medicine Key Laboratory of Sichuan Province, State Key Laboratory of Respiratory Health and Multimorbidity, West China Hospital, Sichuan University, Chengdu, Sichuan, China

**Keywords:** modified Glasgow Prognosis Score(mGPS), lung cancer, biomarker, immunotherapy, meta-analysis

## Abstract

**Background:**

The modified Glasgow Prognosis Score (mGPS), which considers both inflammatory response and nutritional status, has been linked to the prognosis of various tumors. The relationship between mGPS and non-small cell lung cancer (NSCLC) patients receiving immune checkpoint inhibitors (ICIs) is still a subject of debate. This meta-analysis aims to comprehensively assess the association between mGPS and survival in NSCLC treated with ICIs.

**Methods:**

A thorough review of studies from PubMed, Web of Science, Scopus, and Embase was conducted up to June 4, 2024. Fixed-effect or random-effect models were employed, combining hazard ratios (HRs) and 95% confidence intervals (CI), to assess the prognostic value of mGPS for OS and PFS in patients with NSCLC receiving immunotherapy.

**Results:**

A total of 1,022 patients from 11 studies were recruited. Combined results showed that mGPS elevation was significantly associated with poor OS (HR = 1.63, 95%CI: 1.42-1.87, P < 0.01) and PFS (HR = 1.71, 95%CI: 1.31-2.24, P < 0.01). Subgroup analysis and sensitivity analysis further determined the predictive effect of elevated mGPS on OS and PFS deterioration in NSCLC patients receiving immunotherapy.

**Conclusion:**

mGPS can be used as a good noninvasive biomarker to demonstrate prognostic and clinical significance in patients with NSCLC undergoing immunotherapy.

**Systematic review registration:**

http://www.crd.york.ac.uk/prospero/ PROSPERO, identifier CRD42023432661.

## 1 Introduction

Lung cancer ranks among the top causes of cancer-related mortality globally (1). NSCLC is the most prevalent form of lung cancer, comprising 85% of all diagnosed cases (2). In recent years, significant advancements have been made in the treatment of NSCLC. However, the survival rate for metastatic NSCLC is extremely low, with a 5-year survival rate of only about 4% (3). In recent years, immune checkpoint inhibitors (ICIs) have made remarkable strides as a pivotal therapy for treating NSCLC, particularly in advanced stages, bringing new hope to patients (4–7). However, there is significant heterogeneity and individual variation in response to immunotherapy among patients. While some benefit from long-term survival (8, 9), others do not respond to treatment and may experience disease progression (10) or immune-related adverse reactions (11). Identifying new and effective prognostic markers for NSCLC immunotherapy is crucial for making accurate treatment decisions and improving survival outcomes for lung cancer patients.

Previous studies have suggested that the expression level of PD-L1 in tumor cells (12), tumor mutation burden (TMB) (13), microsatellite instability (MSI) (14, 15), and tumor-specific antigens (TSAs) (16) can serve as biomarkers to predict the efficacy of immunotherapy for lung cancer. However, in clinical practice, these methods face several challenges, including high costs, limited tissue sample availability, and the invasiveness of procedures (17).

Previous studies have established that inflammation plays a pivotal role in the initiation and progression of tumors. It is a defining characteristic of malignant tumors and a critical factor in shaping the tumor microenvironment. Tumor cells can trigger inflammation by attracting and activating inflammatory cells within the tumor microenvironment. This process generates reactive oxygen species, leading to protein and DNA damage, ultimately contributing to tumor development and angiogenesis (18). With the continuous in-depth research on the inflammatory response in the tumor microenvironment, more and more inflammatory indicators have been used as good biomarkers to indicate the prognosis and survival outcomes of various malignancies in clinical practice (19–22). In recent years, it has been found that malnutrition is also associated with the prognosis of cancer patients. The incidence of malnutrition among patients with malignant tumors is high, with 40-80% of cancer patients experiencing malnutrition, and 20% of cancer patients dying directly from it (23, 24). The modified Glasgow Outcome score, based on C-reactive protein (CRP) and albumin concentrations, reflects the systemic inflammatory response and nutritional status of patients (25, 26).

mGPS has been widely recognized as an effective prognostic marker, particularly in relation to inflammation and nutritional status in cancer patients. Before the era of ICIs, mGPS was primarily used to assess prognosis in various cancer types, as demonstrated in previous studies (27). Moreover, mGPS has also shown prognostic significance in immunotherapy of multiple types of cancer (28), especially for treatment response and survival outcomes. These findings suggest that mGPS may be a promising marker across both eras and diverse cancer types. However, its prognostic value in NSCLC patients receiving immunotherapy remains inconclusive. Therefore, we aim to explore the application of mGPS in NSCLC immunotherapy through this meta-analysis to contribute to clinical practice.

## 2 Materials and methods

### 2.1 Protocol and registration

This meta-analysis was performed following the 2020 guidelines for Preferred Reporting Items for Systematic Reviews and Meta-Analyses (PRISMA) (29). The International Prospective Register of Systematic Reviews (PROSPERO) registration number for this study is CRD42023432661.

### 2.2 Literature retrieval strategy

We searched the PubMed, Web of Science, Embase, and Scopus databases for eligible studies published up to June 2, 2024. Two independent researchers (JW and HW) conducted the literature search and screening process. Any disagreements were resolved through team consensus. The following terms were used in the search process: Lung Neoplasms, immunotherapy, immune checkpoint inhibitors, nivolumab, pembrolizumab, atezolizumab, avelumab, durvalumab, ipilimumab, tremelimumab, modified Glasgow Prognostic Score. References to the retrieved literature were also carefully reviewed to ensure compliance with inclusion requirements. The detailed search strategy is presented in **Supplementary File S1**.

### 2.3 Inclusion and exclusion criteria for literature

According to PICO principles, the inclusion criteria are as follows:1) Patients with pathologically confirmed advanced lung cancer receiving immunotherapy. 2) CRP and serum albumin levels were available to estimate mGPS. 3) The mGPS scores of 0, 1, and 2 were defined as follows: an elevated CRP level (>1 mg/dl) combined with hypoalbuminemia (<3.5 g/dl) was assigned an mGPS of 2; an elevated CRP level without hypoalbuminemia was assigned an mGPS of 1; and a normal CRP level was assigned an mGPS of 0. 4) The outcome measures included OS, PFS. 5) Randomized controlled trial (RCT) or observational study.

The exclusion criteria are as follows: 1) Case reports, editorials, letters, review literature and conference abstracts without detailed data. 2) Repeated or overlapping data. 3) The results for the NSCLC or immunotherapy groups could not be separated 4) Articles not written in English.

### 2.4 Literature screening and data extraction

All the documents retrieved from the databases were imported into Endnote, and duplicates were first excluded. The titles and abstracts of the remaining articles were meticulously reviewed by two researchers (JW and HW) to exclude irrelevant studies, and full-text versions of potentially eligible studies were further evaluated for inclusion. If there is a disagreement during the process, team discussion with another researcher (RY) was performed to reach a final decision. The extracted data included the first author, publication year, study location, sample size, age, sex, tumor histology (squamous cell carcinoma/non-squamous cell carcinoma), type of ICI, treatment line, comparison of mGPS, HRs and 95% CI. For HR, we prioritized results from multivariate analyses rather than univariate analyses, unless multivariate analyses were unavailable.

### 2.5 Quality assessment

We assessed the quality of the included studies using the Newcastle-Ottawa Scale (NOS), which evaluates three aspects: selection, comparability, and outcome assessment. In the selection evaluation, we assessed the representativeness of the study sample, clearly outlining the requirements for sample sources and inclusion criteria. For the comparability, we evaluated whether the studies controlled for both major and additional confounding factors. The outcome/exposure assessment was based on the objectivity and reliability of the methods used, as well as how loss to follow-up or potential bias in exposure assessment was managed. Studies scoring higher than 6 were classified as high-quality and included in this meta-analysis (30).Any differences are resolved through discussion until an agreement is reached. The results of the quality assessment for each individual study can be seen in **Supplementary File S2**.

### 2.6 Statistical analysis

HR was combined with 95%CI to determine the association between mGPS and OS or/and PFS in NSCLC patients receiving immunotherapy. Q test, I^2^ test and forest plots were used to evaluate the heterogeneity of enrolled studies. The random effects model was used for meta-analysis if the heterogeneity was significant (p<0.10 or I^2^>50%). Otherwise, the fixed effect model was employed. If variables were separated into more than two levels, the results from different levels were merged using a fixed-effect model for further analysis, and Tierney’s methods were applied to estimate the HRs if they were not provided directly (31, 32). We further performed subgroup analysis and sensitivity analysis to explore the sources of heterogeneity. Subgroup analyses were conducted based on study regions, the use of ICIs, and different treatment regimens. For the treatment regimen, “monotherapy” refers to patients who received ICIs as the sole treatment in their current line of therapy. “Combination” refers to patients treated with both chemotherapy and ICI in the same line of therapy. “Mixed” refers to patients who underwent various treatment approaches within the same line of therapy, with some receiving ICI alone and others receiving a combination of ICI and additional therapies, such as chemotherapy. Sensitivity analysis was performed by deleting one study at a time to review individual documents one by one to identify those with the greatest source of heterogeneity. To assess publication bias, a funnel plot and Egger’s test were utilized (33). If significant publication bias was detected, the trim and fill method was applied to adjust the findings (34). Two-tailed P values of less than 0.05 were considered significant. All of the above analyses were performed using R (version 4.3.2).

## 3 Results

### 3.1 Literature search results and characteristics

**Figure 1:**
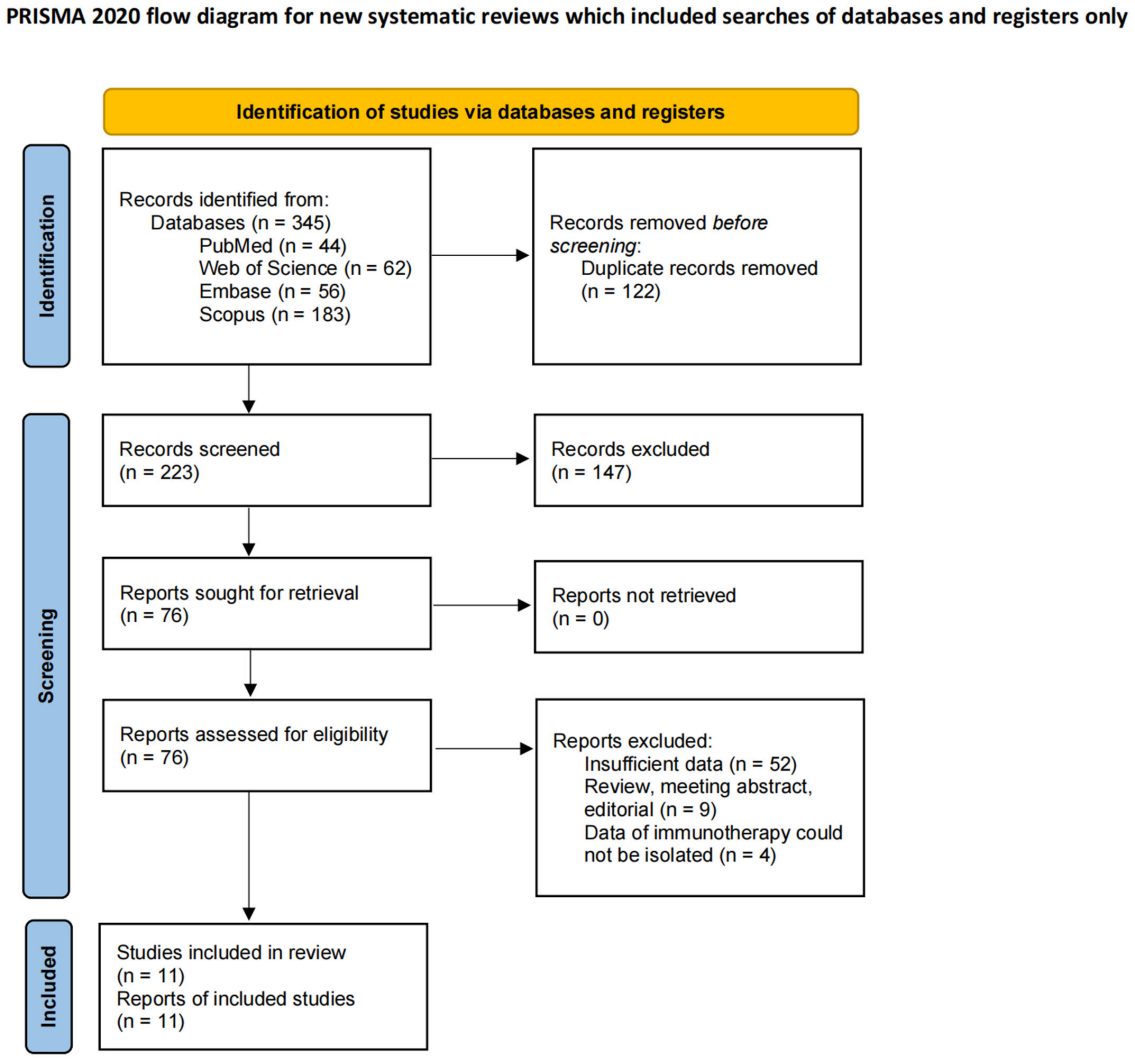
The detailed PRISMA flowchart for the literature screening.

According to the relevant search terms and search strategies, 345 preliminary literatures were obtained in the computer database. After the preliminary de-duplicate, 223 records were selected, and the eligibility of 76 studies was further evaluated through careful review of the full text. Eleven retrospective studies were finally included in the meta-analysis (35–45). The detailed PRISMA flowchart for the literature screening is shown in **Figure 1**, and the checklist is shown in **Supplementary File S3**.

The main characteristics of selected cohorts are summarized in **Table 1**. A total of 1,022 patients from 11 studies were recruited and analyzed. All included studies were retrospective observational cohorts with varying sample sizes ranging from 24 to 304. The patients all had advanced lung cancer, with the highest proportion being elderly males. The ICIs included in the study included nivolumab, pembrolizumab, atezolizumab, camrelizumab and ipilimumab. Six of the studies were conducted in Asian countries and the remaining five were conducted in non-Asian countries. Information on NOS scores is also listed in **Table 1**, and all included studies of high quality. Of the included studies, nine involved patients treated with monotherapy, one involved patients treated with combination therapy, and one involved patients treated with a mixed regimen (**Table 1**).

**Table 1:** Baseline characteristics of included studies.

Study	Region	Sample size	Median age	Sex (Male/Female)	Histology (Sq/NSq)	ICI type	Treatment line (1/more)	Treatment regimen	Outcomes	NOS
Naqash2018	USA	87	64	56/31	39/48	N	0/87	Monotherapy	OS	6
Matsubara2020	Japan	24	64.5	17/7	4/20	A	0/24	Monotherapy	OS	8
Ali2021	China	73	54	51/22	23/50	N, P, A, C	4/69	Monotherapy	OS, PFS	6
Araki2021	Japan	113	68.5	87/26	47/57	N	0/113	Monotherapy	OS	6
Freitas2021	Portugal	77	65	55/22	22/55	N, P	13/64	Monotherapy	OS, PFS	6
Ogura2021	Japan	34	77	29/5	11/23	P, A	34/0	Combination	OS, PFS	6
Takamori2021	Japan	304	66	242/62	74/230	N, P, A	56/248	Monotherapy	OS, PFS	7
Diker2022	Turkey	102	66.5	89/13	36/66	N, P, I	47/55	Mixed	OS, PFS	6
Tanaka2023	Japan	51	79.3	40/11	12/36	N, P	27/24	Monotherapy	OS, PFS	7
Madeddu2023	Italy	74	69.3	54/20	17/57	N,P	42/32	Monotherapy	OS, PFS	7
Olgun2023	North Cyprus	83	66	73/10	32/51	N, P, I	41/42	Monotherapy	OS, PFS	7

Sq, squamous carcinoma; NSq, non-squamous carcinoma; ICI, immune checkpoint inhibitors; USA, the United States of America; N, nivolumab; P, pembrolizumab; A, atezolizumab; C, camrelizumab; I, ipilimumab; OS, overall survival; PFS, progression-free survival.

*Monotherapy refers to patients who received immune checkpoint inhibitors (ICI) alone as the sole treatment in their current line of therapy. Combination refers to patients who were treated with both chemotherapy and immune checkpoint inhibitors in their current line of therapy. Mixed refers to patients in the study who received various treatment approaches within the same line of therapy, including some treated with immune checkpoint inhibitors (ICI) alone and others treated with a combination of ICI and additional therapies, such as chemotherapy.

### 3.2 Prognostic impact of mGPS on survival outcomes in advanced lung cancer patients treated with immunotherapy

**Figure 2:**
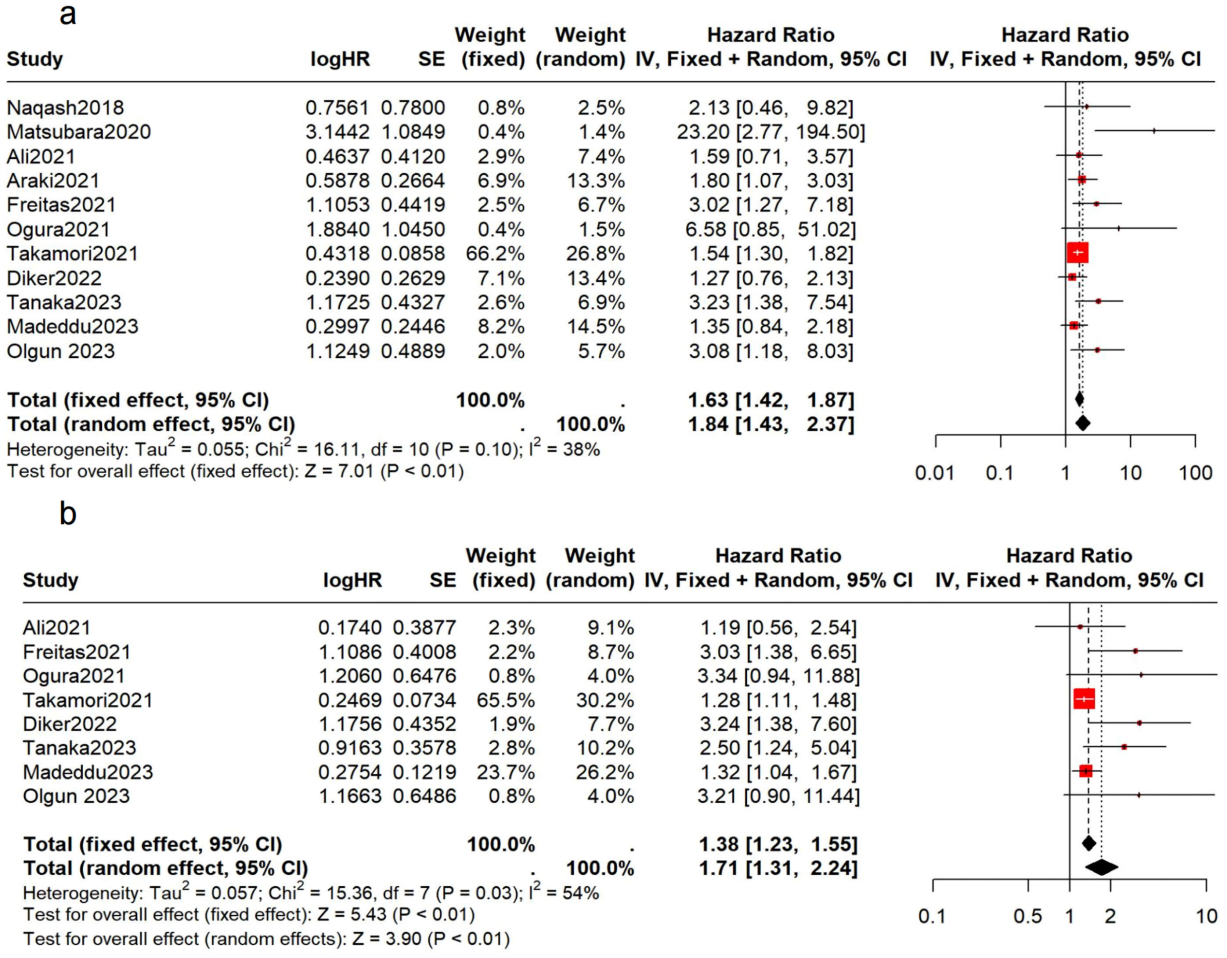
Association between the pretreatment mGPS and survival in NSCLC patients. **(A)** the forest plot of OS; **(B)** the forest plot of PFS.

Preliminary analysis of 1,022 patients from 11 studies showed that patients with elevated mGPS prior to immunotherapy had poorer OS and PFS (OS: HR = 1.63, 95% CI: 1.42-1.87, p < 0.01, I² = 38%, p = 0.10; PFS: HR = 1.71, 95% CI: 1.31-2.24, p < 0.01, I² = 54%, p = 0.03) (**Figure 2**). The I² values indicate low to moderate heterogeneity, with non-significant p-values suggesting that the observed heterogeneity might be due to random variation.

### 3.3 Subgroup analysis

To pinpoint potential sources of heterogeneity, we conducted subgroup analyses based on study region, immunotherapy protocol and treatment regimens. (**Table 2** and **Supplementary Figure S1**).

**Table 2:** Results of the primary analysis and subgroup analyses.

Subgroup	Number of studies	Association		Heterogeneity		Difference
		HR (95%CI)	p	I^2^	p	p
OS						
**Overall**	**11**	**1.63 (1.42-1.87)**	**<0.01**	**38%**	**0.10**	
**Region**						**0.46**
Asia	6	2.10 (1.37-3.22)	<0.01	54%	0.05	
Non-Asia	5	1.70 (1.17-2.46)	<0.01	24%	0.26	
**ICI**						**0.04**
Anti-PD1	5	1.90 (1.36-2.65)	<0.01	13%	0.33	
Anti-PD-L1	1	23.20 (2.77-194.50)	/	/	/	
Mixed^a^	5	1.58 (1.27-1.96)	<0.01	10%	0.35	
**Treatment regimen**						**0.17**
Monotherapy	9	1.92 (1.45-2.55)	<0.01	40%	0.10	
Combination	1	6.58 (0.85-51.02)	/	/	/	
Mixed^b^	1	1.27 (0.76-2.13)	/	/	/	
PFS						
**Overall**	**8**	**1.71 (1.31- 2.24)**	**<0.01**	**54%**	**0.03**	
**Region**						**0.34**
Asia	4	1.58 (1.05-2.37)	0.03	45%	0.14	
Non-Asia	4	2.25 (1.23-4.12)	<0.01	65%	0.03	
**ICI**						**0.84**
Anti-PD1	3	1.96 (1.10-3.49)	0.02	68%	0.04	
Mixed^a^	5	1.82 (1.13-2.92)	0.01	52%	0.08	
**Treatment regimen**						**0.13**
Monotherapy	6	1.51 (1.18-1.92)	<0.01	47%	0.09	
Combination	1	3.34 (0.94-11.88)	/	/	/	
Mixed^b^	1	3.24 (1.38-7.60)	/	/	/	

HR, hazard ratio; CI, confidence interval; OS, overall survival; ICI, immune checkpoint inhibitor; PD1, programmed death 1; PD-L1, programmed death ligand 1; PFS, progression-free survival.

*Mixed^a^ refers to the patients who received monotherapy with ICIs, but the type varied: some were treated with anti-PD-1 antibody, while the others received anti-PD-L1 therapy.

*Monotherapy refers to patients who received immune checkpoint inhibitors (ICI) alone as the sole treatment in their current line of therapy. Combination refers to patients who were treated with both chemotherapy and immune checkpoint inhibitors in their current line of therapy. Mixed^b^ refers to patients in the study who received various treatment approaches within the same line of therapy, including some treated with immune checkpoint inhibitors (ICI) alone and others treated with a combination of ICI and additional therapies, such as chemotherapy.

. For OS, we observed that the effect size differences between different regions and treatment regimens were not significant. The effects of different ICI types on OS were significantly different, suggesting that ICI types might be a source of heterogeneity. This difference may be due to the differences in biological mechanisms of various ICI, or the differences in patient characteristics in different studies, such as treatment line, tumor mutation load and PD-L1 expression level. These variables have not been uniformly measured in existing studies, and future subgroup analyses should be more refined to further identify potential sources of heterogeneity. However, for PFS, subgroup analyses based on regions, treatment regimens, and ICI types did not reveal any significant differences. This lack of variation suggests that further exploration is needed to better understand the potential factors influencing PFS in these subgroups. In addition, further studies are needed to explore other factors that may influence heterogeneity.

### 3.4 Sensitivity analysis

**Figure 3:**
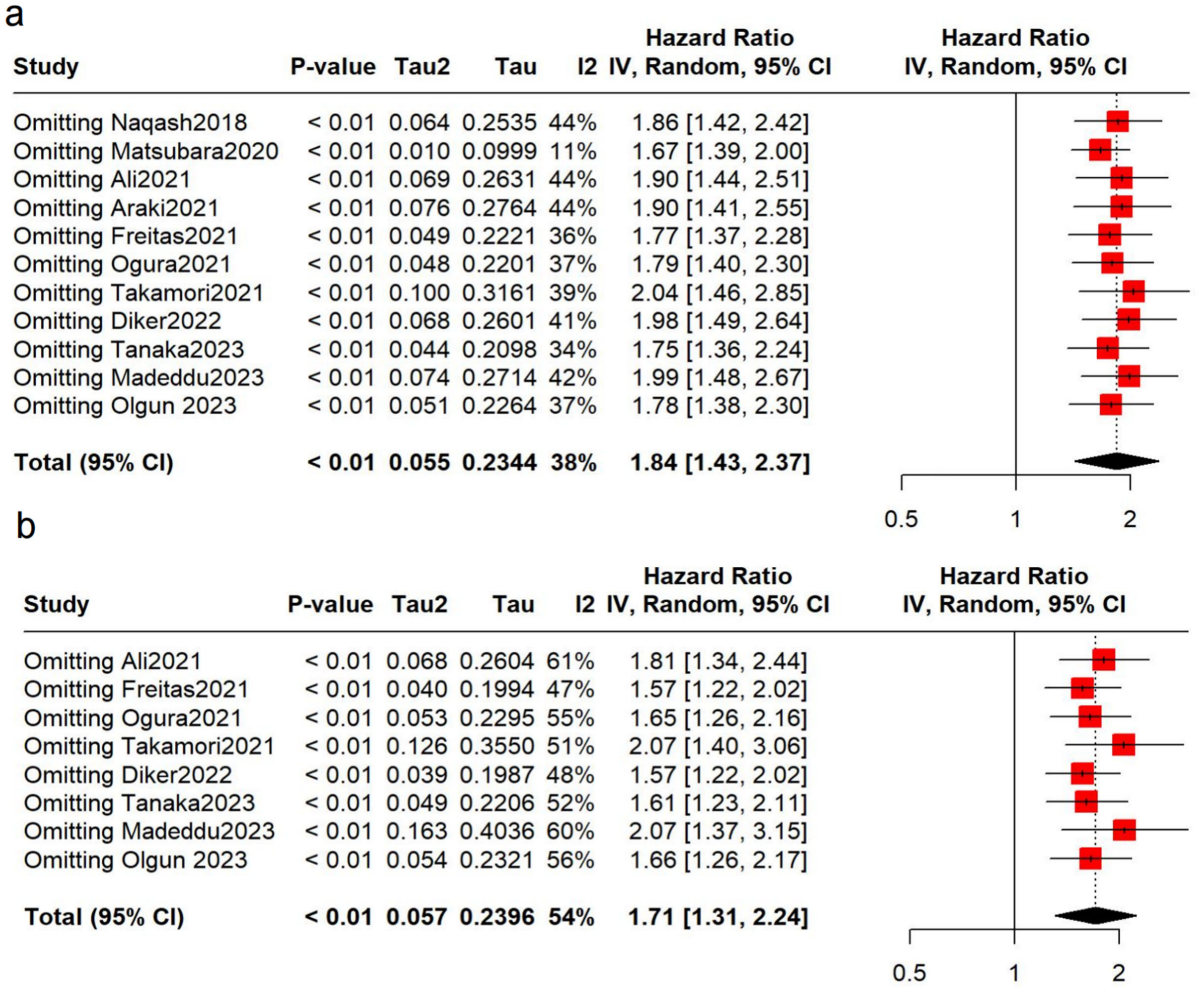
Sensitivity analyses. **(A)** analysis for OS; **(B)** analysis for PFS.

To verify the robustness of the results, sensitivity analyses were conducted. No over-representation of study was observed, and the pooled results remained robust (**Figure 3**). However, after removing the Matsubara study (34), we found that the heterogeneity decreased significantly (OS: I^2^ = 11%), suggesting that this may be a main contributor to the heterogeneity.

### 3.5 Publication bias

**Figure 4:**
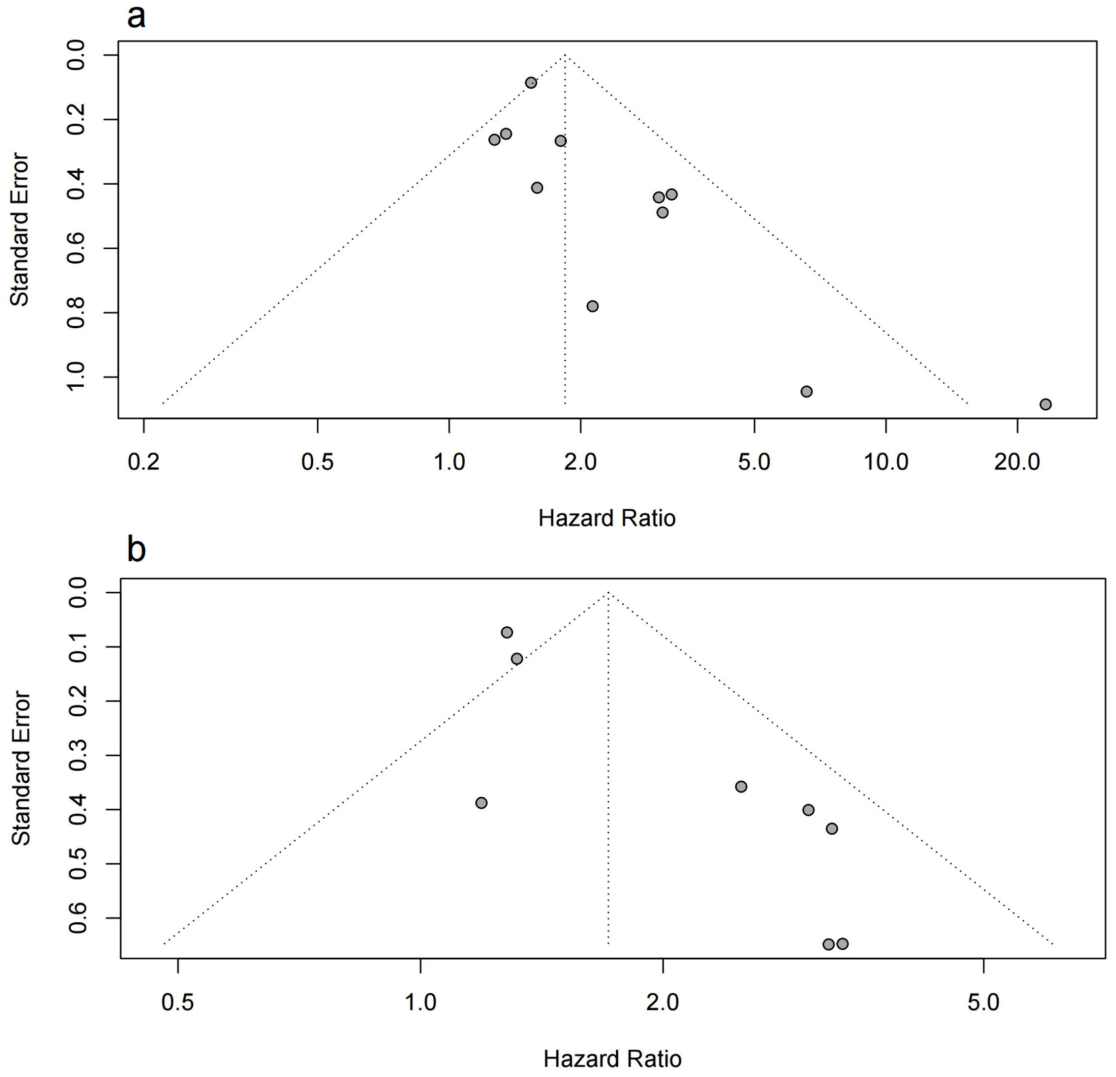
Funnel plot of the meta-analysis. **(A)** the funnel plot for OS; **(B)** the funnel plot for PFS.

Asymmetry was observed in the funnel plot, indicating a potential publication bias (**Figure 4**). This was further supported by the Egger test results, which were statistically significant for both OS and PFS (p < 0.05). The asymmetry could be due to the selective publication of studies with positive results, which is a common issue in meta-analyses.

To account for potential publication bias, we conducted Trim-and-Fill analyses, which identified missing studies that were likely unpublished due to non-significant findings. After adjusting for these missing studies, the results remained robust and consistent with the original findings, reinforcing the reliability of our conclusions. The detailed results of this analysis can be found in **Supplementary Figures S2** and **S3**.

In addition, we acknowledge that publication bias, even after adjustment, may still have a residual effect. We recommend that future studies incorporate unpublished data to further mitigate this limitation.

## 4 Discussion

Lung cancer has an extremely high disease burden characterized by rapid progression and poor survival. In recent years, the emergence of immunotherapy has brought new hope to patients, and many patients have benefited from immunotherapy (46). However, due to the high price and uncertain efficacy of immunotherapy, and some patients have adverse consequences such as drug resistance (47) or immune-related adverse events(irAEs) (48), it is urgent to find non-invasive prognostic biomarkers of immunotherapy to guide clinical medication and screen suitable patients.

This is the first meta-analysis to include 11 original studies with a total of 1,022 patients and to incorporate immunotherapy outcomes. The analysis showed that higher pre-medication mGPS in lung cancer patients receiving immunotherapy was associated with a poorer prognosis, with significantly lower OS and PFS. These findings indicate that mGPS could be a valuable biomarker for guiding lung cancer immunotherapy in clinical practice, contributing to precision medicine. Subgroup analysis and sensitivity analysis demonstrated the robustness of our results on different parameters.

Previous studies demonstrated the clinical utility of mGPS in non-immunotherapy recipients with different tumors, and our results were similar to theirs (27, 28). The consistency of our findings underscores the utility of mGPS as a prognostic biomarker for NSCLC immunotherapy, further validating its broad applicability and reinforcing the notion that systemic inflammation plays a critical role in cancer prognosis, regardless of the era of therapy.

Inflammation is considered one of the hallmarks of cancer (49), inflammatory cells and cytokines are key components of the tumor microenvironment (50).Activated inflammatory cells and their mediators cause mutations in epithelial cells through the production of reactive oxygen species and active nitrogen intermediates, leading to the development of tumors (51). The incidence of malnutrition in patients with malignant tumors is high, and 40-80% of cancer patients are malnourished (24). Therefore, Forrest and colleagues first (52) proposed the Glasgow Prognostic Score (GPS) in combination of CRP and serum albumin, and confirmed its prognostic value in NSCLC, particularly when considering the clinical stage. However, hypoalbuminemia alone is rare, and few patients exhibit hypoalbuminemia when CRP levels are normal (53). Consequently, an improved Glasgow Prognostic Scoring system, mGPS, was developed. While other inflammation or nutrition-based markers, such as the neutrophil-lymphocyte ratio (NLR) and prognostic nutritional index (PNI), have been investigated for their prognostic value in various cancers, mGPS, appeared to be more effective, comprehensively reflecting both systemic inflammatory response and nutritional status (54, 55). Moreover, it has low cost, high repeatability and clear cut-off value, and can be widely used in clinical practice.

The mechanism behind mGPS also needs to be explored and understood. Previous study indicated that CRP is a key protein in acute phase reactions. Its blood levels have been widely used as a minimally invasive marker for persistent inflammatory responses, including those associated with cancer (56). Research has shown that elevated serum CRP is induced by IL-6, which in turn promotes resistance to ICIs. Additionally, high CRP levels are associated with low levels of CD4+ T cells, which partly explains the poor prognosis of immunotherapy in patients with elevated CRP (57). Decreased albumin concentrations in the course of cancer usually indicate malnutrition and cachexia (58). Moreover, albumin can create immunosuppressive microenvironments by activating prostaglandin E2, which affects immune function and reduces immune cell activity. This confirms that albumin levels are linked to immunotherapy outcomes (59).

There are some limitations to our study that need to be addressed by further research in the future.

First of all, as this meta-analysis primarily included retrospective observational studies, certain limitations are inevitable. While we minimized the impact of confounders through subgroup and sensitivity analyses, we could not fully eliminate selection and information bias, limiting the results to correlations rather than establishing causation. Additionally, the quality and consistency of retrospective data may contribute to heterogeneity and bias. To better address heterogeneity, future research should mitigate the limitations of observational studies by employing rigorous prospective designs, with a particular focus on controlling confounding factors and selection bias. Larger sample sizes and standardized immunotherapy regimens in these prospective studies will allow for a more precise assessment of the prognostic value of the modified Glasgow Prognostic Score (mGPS) in NSCLC, while reducing the biases inherent in current observational study designs. The second limitation is the presence of publication bias. We addressed this by performing Egger’s test and analyzing the funnel plot to assess the potential for publication bias in our meta-analysis. Both methods indicated some degree of publication bias, which is a common issue in meta-analyses involving observational studies. We also performed Trim-and-Fill analyses to assess the robustness of our results. The results indicated that the adjusted results remained consistent with the original findings. However, some degree of publication bias may still be present. We acknowledge this limitation and recommend that future research incorporate unpublished data and conduct additional analyses to further address this issue. Third, due to the lack of the original data of the included studies, we could not conduct more detailed analyses in the next step, nor could we obtain the impact of dynamic changes in mGPS on the prognosis of immunotherapy. Future studies should explore the prognostic value of changes in mGPS in the course of anti-tumor immunotherapy. Finally, since most of included studies only involved patients receiving ICIs monotherapy, we were unable to compare the prognostic outcomes of combination immunotherapy versus monotherapy. Further research is needed to explore the prognostic value of mGPS in combination immunotherapy.

## 5 Conclusion

Our study confirms that a higher mGPS is associated with poorer outcomes in lung cancer patients receiving immunotherapy. Patients with higher mGPS exhibit shorter long-term survival and progression-free survival. More large-scale prospective multicenter clinical studies are needed in the future to confirm this finding.

## Data Availability

The original contributions presented in the study are included in the article/**Supplementary Material**. Further inquiries can be directed to the corresponding author.
