# Supplementary figures and images for: Prognostic significance of the modified Glasgow Prognostic Score in NSCLC patients undergoing immune checkpoint inhibitor therapy: a meta-analysis
Source: Front Oncol. 2024 Oct 11;14:1449853. doi: 10.3389/fonc.2024.1449853 (PMC11502296; doi:10.3389/fonc.2024.1449853)

a

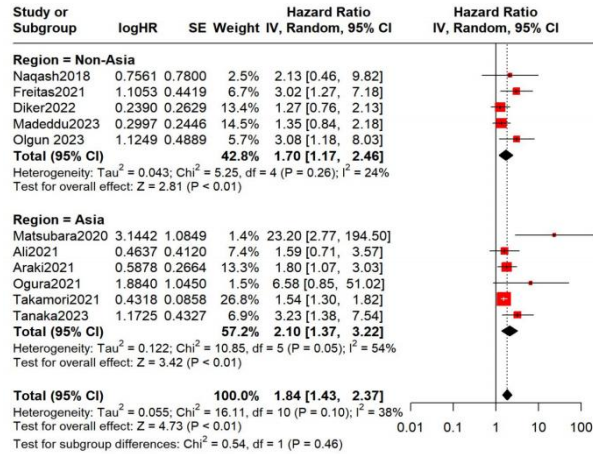

d

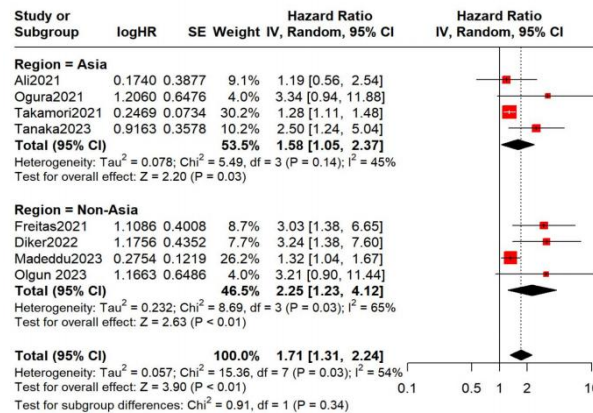

b

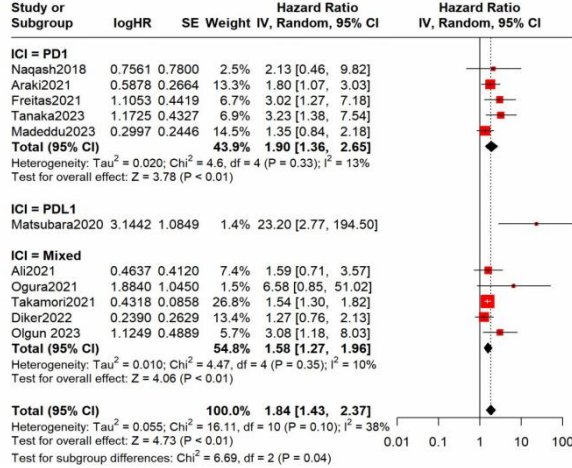

e

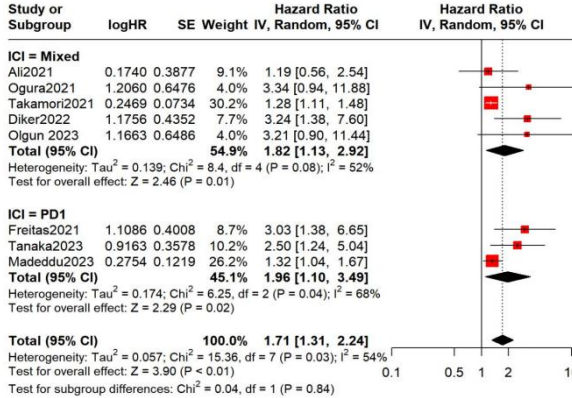

c

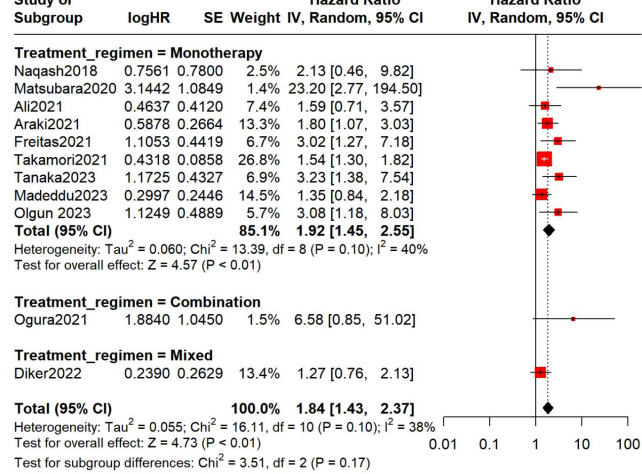

f

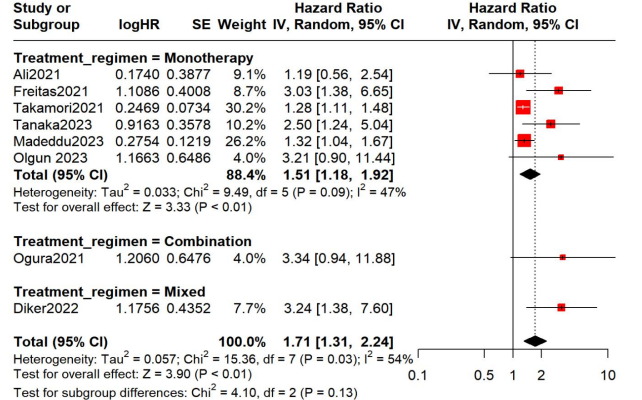

Supplement: Supplementary file 1 [file Image1.pdf]

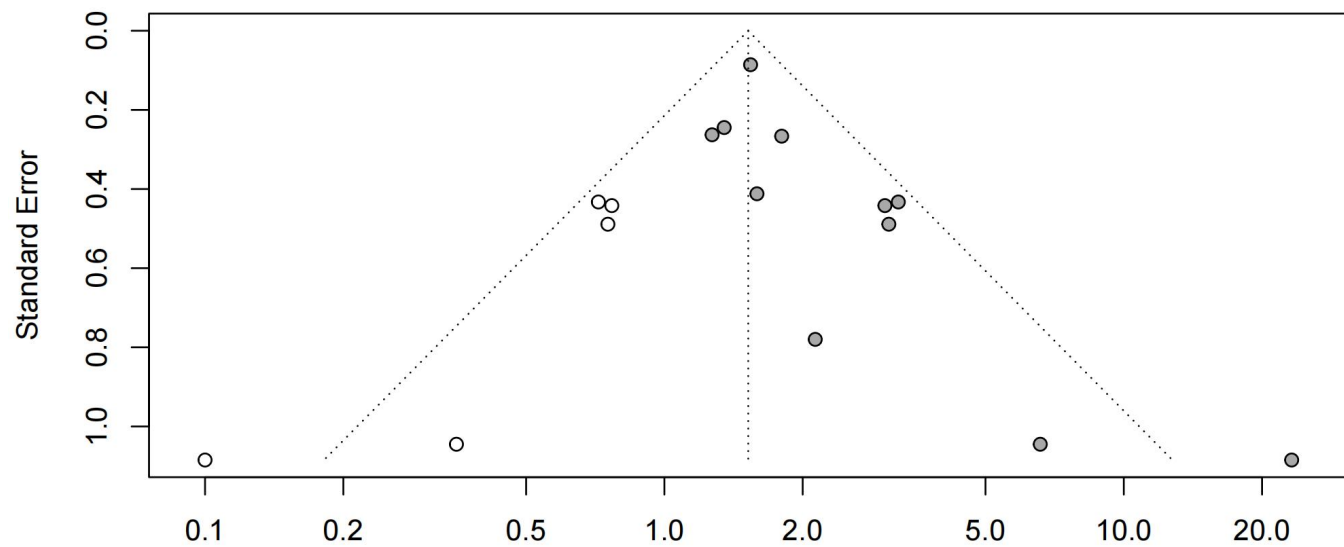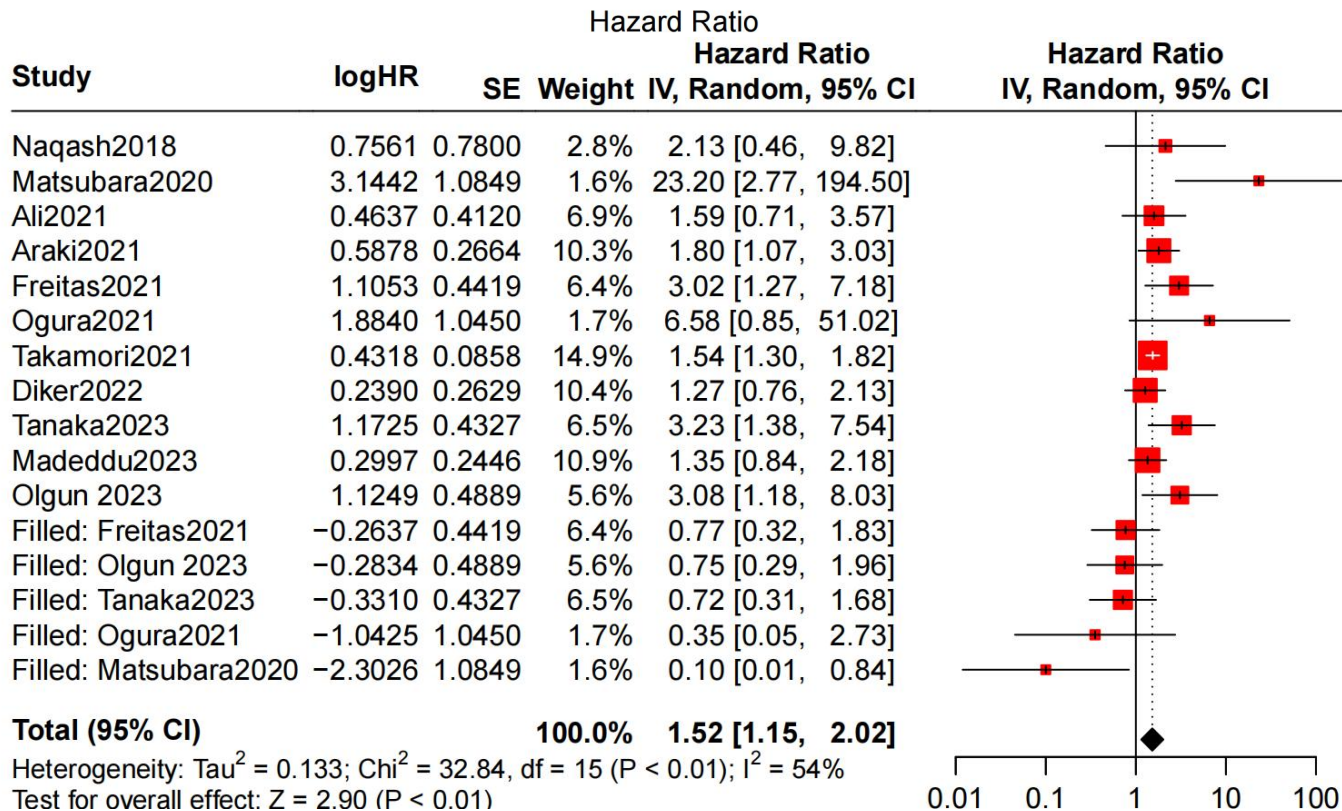

Supplement: Supplementary file 2 [file Image2.pdf]

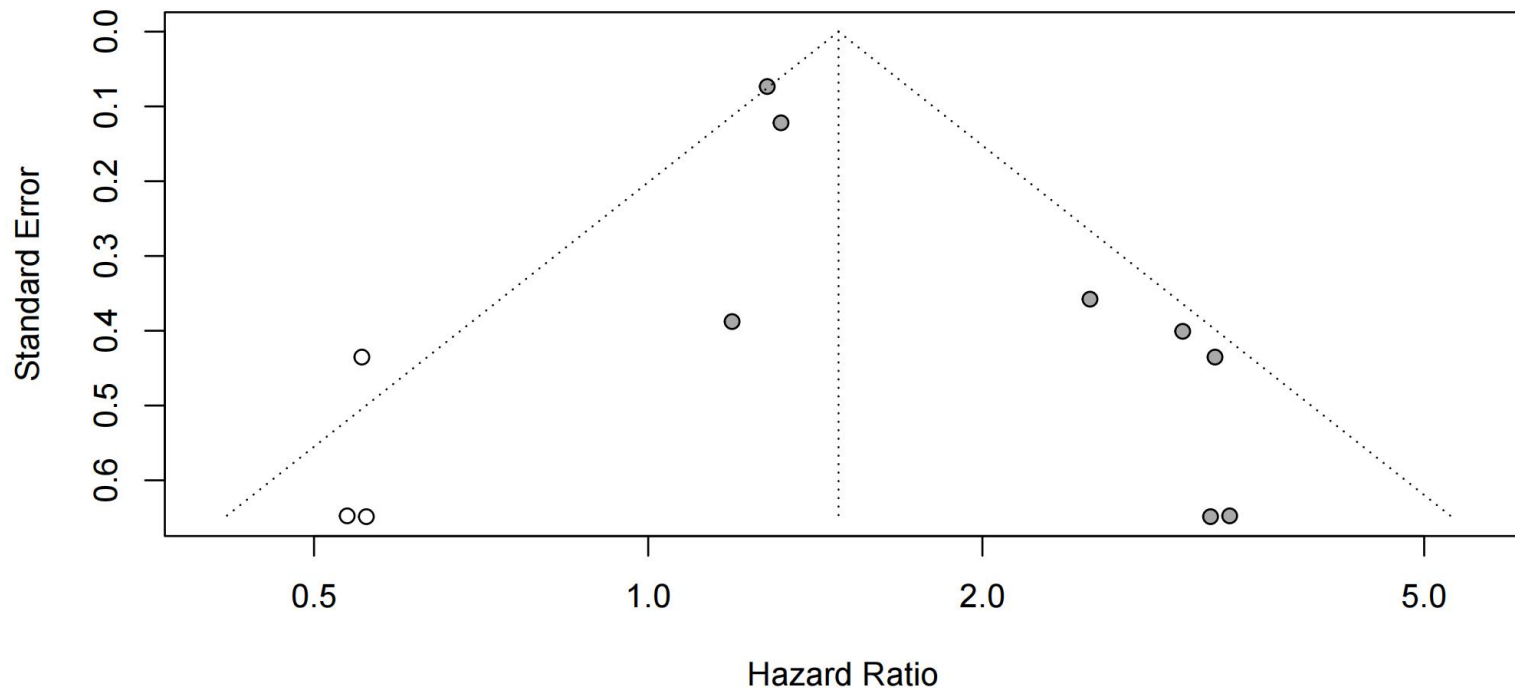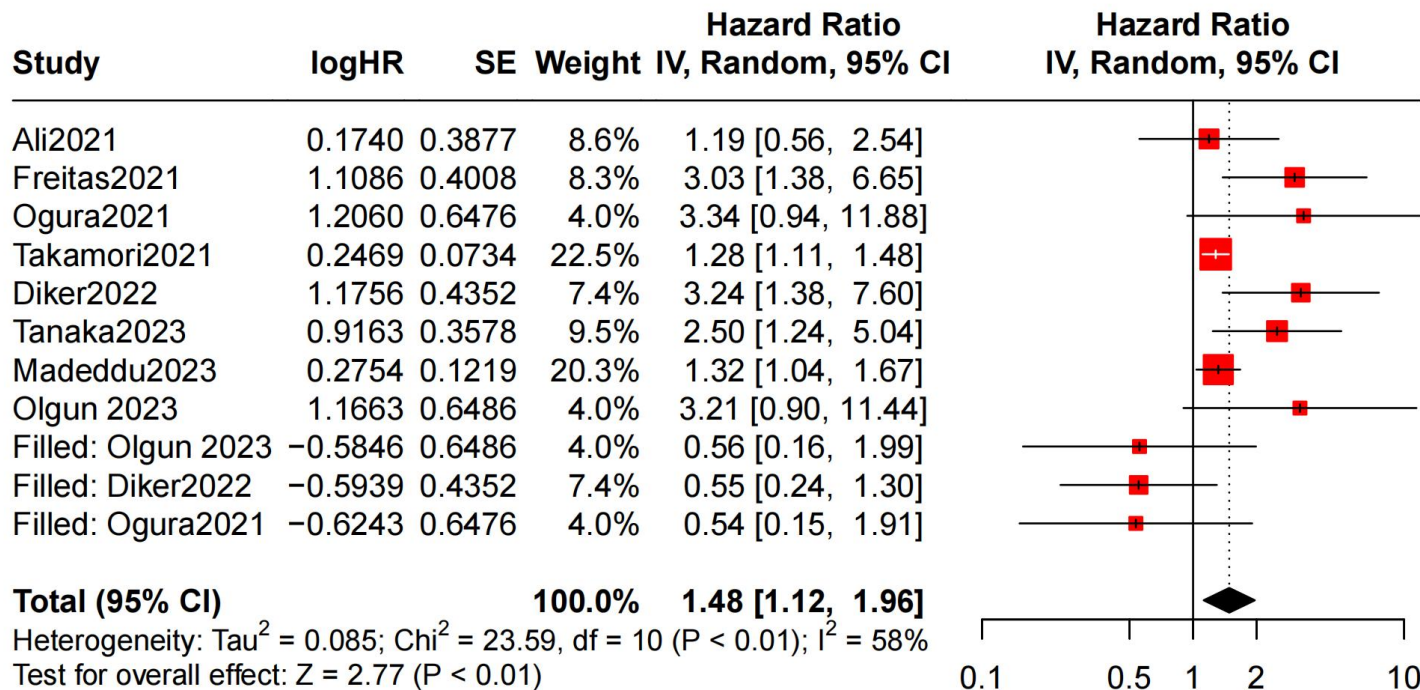

Supplement: Supplementary file 3 [file Image3.pdf]
