# Supplementary material for: Prognostic significance of the modified Glasgow Prognostic Score in NSCLC patients undergoing immune checkpoint inhibitor therapy: a meta-analysis
Source: Front Oncol. 2024 Oct 11;14:1449853. doi: 10.3389/fonc.2024.1449853 (PMC11502296; doi:10.3389/fonc.2024.1449853)
Supplement: Supplementary file 4 [file Table1.docx]

**Supplementary file 1. Search strategies**

**PubMed**

#1:

Lung Neoplasms[Mesh] OR Pulmonary Neoplasms OR Neoplasms, Lung OR Lung Neoplasm OR Neoplasm, Lung OR Neoplasms, Pulmonary OR Neoplasm, Pulmonary OR Pulmonary Neoplasm OR Lung Cancer OR Cancer, Lung OR Cancers, Lung OR Lung Cancers OR Pulmonary Cancer OR Cancer, Pulmonary OR Cancers, Pulmonary OR Pulmonary Cancers OR Cancer of the Lung OR Cancer of Lung

#2:

immunotherapy[Mesh] OR immunotherapies

#3:

immune checkpoint inhibitors[Mesh] OR Checkpoint Inhibitors, Immune OR Immune Checkpoint Inhibitor OR Checkpoint Inhibitor, Immune OR Immune Checkpoint Blockers OR Checkpoint Blockers, Immune OR Immune Checkpoint Blockade OR Checkpoint Blockade, Immune OR Immune Checkpoint Inhibition OR Checkpoint Inhibition, Immune OR PDL1 Inhibitors OR PD L1 Inhibitors OR PDL1 Inhibitor OR PD L1 Inhibitor OR Programmed Death Ligand 1 Inhibitors OR Programmed Death Ligand 1 Inhibitors OR PD1PDL1 Blockade OR Blockade, PD1PDL1 OR PD 1 PD L1 Blockade OR CTLA4 Inhibitors OR CTLA 4 Inhibitors OR CTLA4 Inhibitor OR CTLA 4 Inhibitor OR Cytotoxic T Lymphocyte Associated Protein 4 Inhibitors OR Cytotoxic T Lymphocyte Associated Protein 4 Inhibitors OR Cytotoxic T Lymphocyte Associated Protein 4 Inhibitor OR Cytotoxic T Lymphocyte Associated Protein 4 Inhibitor OR PD1 Inhibitors OR PD 1 Inhibitors OR PD1 Inhibitor OR Inhibitor, PD1 OR PD 1 Inhibitor OR Programmed Cell Death Protein 1 Inhibitor OR Programmed Cell Death Protein 1 Inhibitors

#4:

nivolumab OR pembrolizumab OR atezolizumab OR avelumab OR durvalumab OR ipilimumab OR tremelimumab

#5:

#2 OR #3 OR #4

#6:

#1 AND #5

#7:

Glasgow Prognostic Score OR GPS OR modified Glasgow Prognostic Score OR mGPS

#8:

#6 AND #7

**Web of Science**

#1:

TS=(Lung Neoplasms OR Pulmonary Neoplasms OR Neoplasms, Lung OR Lung Neoplasm OR Neoplasm, Lung OR Neoplasms, Pulmonary OR Neoplasm, Pulmonary OR Pulmonary Neoplasm OR Lung Cancer OR Cancer, Lung OR Cancers, Lung OR Lung Cancers OR Pulmonary Cancer OR Cancer, Pulmonary OR Cancers, Pulmonary OR Pulmonary Cancers OR Cancer of the Lung OR Cancer of Lung)

#2:

TS=(immunotherapy OR immunotherapies)

#3:

TS=(immune checkpoint inhibitors OR Checkpoint Inhibitors, Immune OR Immune Checkpoint Inhibitor OR Checkpoint Inhibitor, Immune OR Immune Checkpoint Blockers OR Checkpoint Blockers, Immune OR Immune Checkpoint Blockade OR Checkpoint Blockade, Immune OR Immune Checkpoint Inhibition OR Checkpoint Inhibition, Immune OR PDL1 Inhibitors OR PD L1 Inhibitors OR PDL1 Inhibitor OR PD L1 Inhibitor OR Programmed Death Ligand 1 Inhibitors OR Programmed Death Ligand 1 Inhibitors OR PD1PDL1 Blockade OR Blockade, PD1PDL1 OR PD 1 PD L1 Blockade OR CTLA4 Inhibitors OR CTLA 4 Inhibitors OR CTLA4 Inhibitor OR CTLA 4 Inhibitor OR Cytotoxic T Lymphocyte Associated Protein 4 Inhibitors OR Cytotoxic T Lymphocyte Associated Protein 4 Inhibitors OR Cytotoxic T Lymphocyte Associated Protein 4 Inhibitor OR Cytotoxic T Lymphocyte Associated Protein 4 Inhibitor OR PD1 Inhibitors OR PD 1 Inhibitors OR PD1 Inhibitor OR Inhibitor, PD1 OR PD 1 Inhibitor OR Programmed Cell Death Protein 1 Inhibitor OR Programmed Cell Death Protein 1 Inhibitors)

#4:

TS=(nivolumab OR pembrolizumab OR atezolizumab OR avelumab OR durvalumab OR ipilimumab OR tremelimumab)

#5:

#2 OR #3 OR #4

#6:

#1 AND #5

#7:

TS=(Glasgow Prognostic Score OR GPS OR modified Glasgow Prognostic Score OR mGPS)

#8:

#6 AND #7

**Embase**

1 lung cancer.mp. or exp lung cancer/

2 immunotherapy.mp. or exp cancer immunotherapy/ or exp immunotherapy/

3 immune checkpoint inhibitor.mp. or exp immune checkpoint inhibitor/

4 (nivolumab or pembrolizumab or atezolizumab or avelumab or durvalumab or ipilimumab or tremelimumab).mp.

5 (Glasgow Prognostic Score or modified Glasgow Prognostic Score).mp.

6 2 or 3 or 4

7 1 and 5 and 6

**Scopus**

ALL("Glasgow Prognostic Score" OR "GPS" OR "modified Glasgow Prognostic Score" OR "mGPS") AND TITLE-ABS-KEY("Lung Neoplasms" OR "Pulmonary Neoplasms" OR "Neoplasms, Lung" OR "Lung Neoplasm" OR "Neoplasm, Lung" OR "Neoplasms, Pulmonary" OR "Neoplasm, Pulmonary" OR "Pulmonary Neoplasm" OR "Lung Cancer" OR "Cancer, Lung" OR "Cancers, Lung" OR "Lung Cancers" OR "Pulmonary Cancer" OR "Cancer, Pulmonary" OR "Cancers, Pulmonary" OR "Pulmonary Cancers" OR "Cancer of the Lung" OR "Cancer of Lung") AND (TITLE-ABS-KEY("Immunotherapy" OR "immunotherapies") OR TITLE-ABS-KEY("immune checkpoint inhibitors" OR "Checkpoint Inhibitors, Immune" OR "Immune Checkpoint Inhibitor" OR "Checkpoint Inhibitor, Immune" OR "Immune Checkpoint Blockers" OR "Checkpoint Blockers, Immune" OR "Immune Checkpoint Blockade" OR "Checkpoint Blockade, Immune" OR "Immune Checkpoint Inhibition" OR "Checkpoint Inhibition, Immune" OR "PDL1 Inhibitors" OR "PD L1 Inhibitors" OR "PDL1 Inhibitor" OR "PD L1 Inhibitor" OR "Programmed Death Ligand 1 Inhibitors" OR "Programmed Death Ligand 1 Inhibitors" OR "PD1PDL1 Blockade" OR "Blockade, PD1PDL1" OR "PD 1 PD L1 Blockade" OR "CTLA4 Inhibitors" OR "CTLA 4 Inhibitors" OR "CTLA4 Inhibitor" OR "CTLA 4 Inhibitor" OR "Cytotoxic T Lymphocyte Associated Protein 4 Inhibitors" OR "Cytotoxic T Lymphocyte Associated Protein 4 Inhibitors" OR "Cytotoxic T Lymphocyte Associated Protein 4 Inhibitor" OR "Cytotoxic T Lymphocyte Associated Protein 4 Inhibitor" OR "PD1 Inhibitors" OR "PD 1 Inhibitors" OR "PD1 Inhibitor" OR "Inhibitor, PD1" OR "PD 1 Inhibitor" OR "Programmed Cell Death Protein 1 Inhibitor" OR "Programmed Cell Death Protein 1 Inhibitors") OR TITLE-ABS-KEY("nivolumab" OR "pembrolizumab" OR "atezolizumab" OR "avelumab" OR "durvalumab" OR "ipilimumab" OR "tremelimumab"))
