# Supplementary material for: Prognostic significance of the modified Glasgow Prognostic Score in NSCLC patients undergoing immune checkpoint inhibitor therapy: a meta-analysis
Source: Front Oncol. 2024 Oct 11;14:1449853. doi: 10.3389/fonc.2024.1449853 (PMC11502296; doi:10.3389/fonc.2024.1449853)
Supplement: Supplementary file 5 [file Table2.docx]

**Supplementary file 2. The results of the quality assessment for each individual study**

|  | **Selection** | | | | **Comparability** | | **Exposure** | | |  |
| --- | --- | --- | --- | --- | --- | --- | --- | --- | --- | --- |
| Assessment criteria | Case definition adequate | Representativeness  of the cases | Selection of controls | Definition of controls | Comparability based on design or analysis | | Ascertainment of exposure | Same method of ascertainment for cases and controls | Non-response rate | Total |
| Naqash2018 | ● | ● | ● | ● | ○ | ○ | ● | ● | ○ | 6 |
| Matsubara2020 | ● | ● | ● | ● | ● | ● | ● | ● | ○ | 8 |
| Ali2021 | ● | ● | ● | ● | ○ | ○ | ● | ● | ○ | 6 |
| Araki2021 | ● | ● | ● | ● | ○ | ○ | ● | ● | ○ | 6 |
| Freitas2021 | ● | ● | ● | ● | ○ | ○ | ● | ● | ○ | 6 |
| Ogura2021 | ● | ● | ● | ● | ○ | ○ | ● | ● | ○ | 6 |
| Takamori2021 | ● | ● | ● | ○ | ● | ● | ● | ● | ○ | 7 |
| Diker2022 | ● | ● | ● | ● | ○ | ○ | ● | ● | ○ | 6 |
| Tanaka2023 | ● | ● | ● | ● | ● | ○ | ● | ● | ○ | 7 |
| Madeddu2023 | ● | ● | ● | ● | ● | ○ | ● | ● | ○ | 7 |
| Olgun 2023 | ● | ● | ● | ● | ● | ○ | ● | ● | ○ | 7 |
